# Hot Deformation Behavior and Microstructure Evolution of 14Cr ODS Steel

**DOI:** 10.3390/ma11061044

**Published:** 2018-06-20

**Authors:** Qian Zhao, Liming Yu, Zongqing Ma, Huijun Li, Zumin Wang, Yongchang Liu

**Affiliations:** State Key Lab of Hydraulic Engineering Simulation and Safety, School of Materials Science & Engineering, Tianjin University, Tianjin 300354, China; zhaoqiantjums@163.com (Q.Z.); lmyu@tju.edu.cn (L.Y.); zqma@tju.edu.cn (Z.M.); huijun@uow.edu.au (H.L.); Z.wang@tju.edu.cn (Z.W.)

**Keywords:** ODS steel, hot deformation, constitutive equation, microstructure evolution, nanoparticles

## Abstract

Hot deformation tests of 14Cr oxide dispersion strengthened (ODS) steel fabricated by mechanical alloying and hot isostatic pressing (HIP) were performed on a Gleeble-1500D simulator at temperatures ranging from 1050 to 1200 °C with the strain rate range of 0.001^−1^ s^−1^. The relationship between the rheological stress and the deformation condition was studied, and a processing map at the true strain of 0.5 was proposed. Microstructure evolution during the deformation process and the effects of deformation conditions on microstructures were also investigated, as well as the stability of nanoparticles. Results show that the 14Cr ODS steel possesses positive strain rate sensitivity. The flow stress increases with the decrease of deformation temperature and the increase of strain rate. The recrystallization process is promoted by the increase of deformation temperature and the reduction of strain rate. Nanoparticles possess excellent stability during the deformation process and are coherent with the matrix.

## 1. Introduction

Oxide dispersion strengthened (ODS) steel is the potential candidate for the structural material in future nuclear reactors [1,2,3,4,5]. Nanoparticles such as Y_2_O_3_, Y_2_Ti_2_O_7_, Y_2_TiO_5_, Y_4_Al_2_O_9_, Y_3_Al_5_O_12_, and YAlO_3_ play an important role in ODS steel [6,7,8,9]. These nanoparticles are uniformly distributed in the matrix, acting as the pinning points for dislocation movement and grain boundary motion [7]. The stability of nanoparticles has been investigated by many researchers [10,11,12,13,14,15,16,17]. The stable nanoparticles and the efficient pinning effect of them lead to the excellent high temperature and superior irradiation resistance of ODS steel [5,18,19,20]. The improvement of material performance is closely related to the further development of nuclear reactors.

The powder metallurgy method is generally utilized to fabricate ODS steel. The fabrication route includes mechanical alloying and subsequent consolidation. The pre-alloyed powders are mechanically alloyed with the addition of nanosized oxides and other elements. Hot extrusion (HE), spark plasma sintering (SPS), and hot isostatic pressing (HIP) are the frequently used sintering methods for the consolidation of ODS steel [21,22,23,24,25,26,27,28,29,30,31,32]. The final product of ODS steel is a cladding tube, which means that hot or cold working is inevitable during the fabrication process [1,5]. It has been shown by investigation that a cladding tube fabricated by hot extrusion and rolling presents a reduction of creep strength and ductility in the biaxial hoop direction [1,5]. Microstructures with different textures are proven to possess different recrystallization behaviors in ODS steel [33,34]. The anisotropic microstructure obtained via HE always possesses elongated grains, meaning the optimal distribution of texture, which has important influences on the recrystallization behavior [34]. Though the HIPed ODS steel tends to possess an isotropic microstructure, further hot or cold working is still an essential step for the fabrication of the final product [1,26]. Therefore, the workability of the ODS steel needs to be investigated in detail. Knowledge of microstructure evolution during manufacture is beneficial for the practical production.

The deformation behavior and microstructure evolution of different ferritic steels and alloys have been analyzed and discussed by previous researchers [35,36,37,38,39]. Zhang et al. [40] have established the constitutive equation and processing map of 9Cr ODS steel, but the microstructure evolution has not been investigated. For now, research about the hot deformation behavior of 14Cr ODS steel has not been widely reported. Although the annealing behavior and static recrystallization of ODS steel have been investigated by some researchers [10,16], knowledge about the recrystallization caused by deformation is still inadequate.

In this paper, the deformation behavior of 14Cr ODS steel was investigated. The flow behavior of the ODS steel during the deformation process under different deformation conditions was analyzed on the basis of true strain–stress curves. The processing map was drawn according to the dynamic materials model (DMM). The relationship between the microstructure evolution and the deformation conditions was identified. Substructures and nanoparticles in the deformed microstructure are characterized and analyzed as well.

## 2. Experimental Procedure

**Figure 1:**
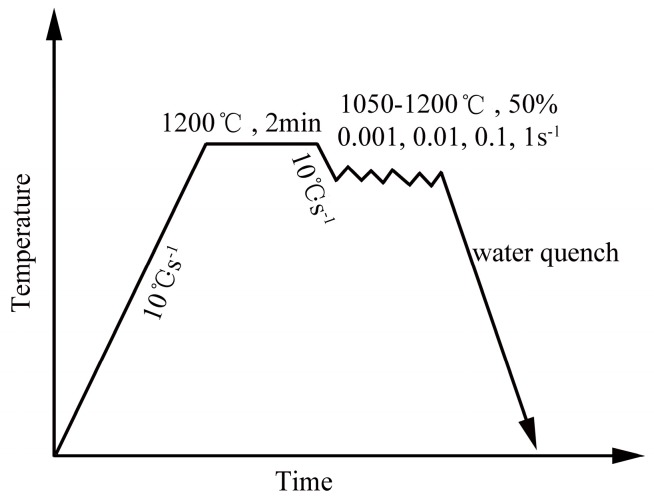
Schematic diagram of hot deformation parameters.

The ODS steel investigated in this work was fabricated by the powder metallurgy method. The pre-alloyed powders with the nominal composition of Fe–14Cr–2W–0.2V–0.07Ta (wt %) were mechanically alloyed with the addition of 0.3 wt % Ti and 0.3 wt % Y_2_O_3_ for 30 h at 400 rpm in a planetary ball mill. The mechanical alloying process was conducted in stainless jars under a high-purity argon atmosphere with the ball-to-powder ratio of 15:1. The obtained powders with composition Fe–14Cr–2W–0.2V–0.07Ta–0.3Ti–0.3Y_2_O_3_ were then consolidated by hot isostatic pressing (HIP) for 3 h at 1150 °C under the pressure of 150 MPa. The as-HIPed ODS steel was then annealed at 1000 °C for 5 h. The columnar samples with the size of Φ 8 × 12 mm were manufactured from the annealed ODS steel. The hot deformation tests were performed on a Gleeble-1500D simulator at temperatures ranging from 1050 to 1200 °C with the internal of 50 °C. The stain rate ranged from 0.001 to 1 s^−1^. After deformation, the samples were subsequently quenched in water. The schematic diagram of the hot deformation parameters is shown in Figure 1.

The deformed microstructure was characterized by optical microscope (OM, Leica DFC 450, Wetzral, Hesse, Germany), scanning electron microscope (SEM, SU1510, Tokyo, Japan), and transmission electron microscope (TEM, JEM-2100F, Tokyo, Japan). Samples for OM and SEM characterization were prepared by the standard metallographic preparation method. The samples were mechanically grinded, polished, and then etched in a solution composed of 5 g copper chloride, 100 mL hydrochloric acid, and 100 mL ethyl alcohol. Thin foil samples were used for the TEM investigation and electropolished in a double-jet electropolishing device at −20 °C. The electrolyte was a mixture consisting of 5% perchloric acid and 95% ethanol.

## 3. Results and Discussion

### 3.1. Hot Deformation Behavior and Processing Map

#### 3.1.1. Strain–Stress Curves

**Figure 2:**
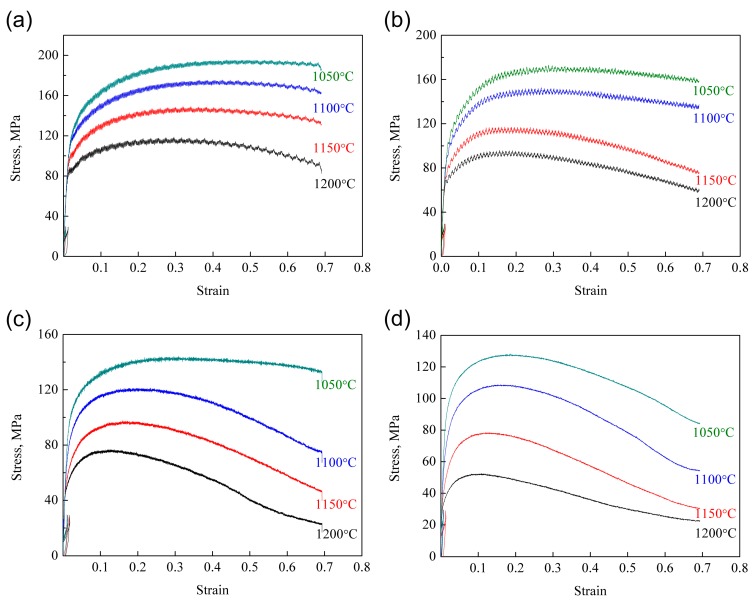
True strain–stress curves of the deformed oxide dispersion strengthened (ODS) samples: (**a**) 1 s^−1^; (**b**) 0.1 s^−1^; (**c**) 0.01 s^−1^; (**d**) 0.001 s^−1^.

The true strain–stress curves of the deformed ODS samples are illustrated in Figure 2. As can be seen from the figure, all the curves possess similar characteristics, which can be attributed to the competing relationship between work hardening and dynamic softening. The flow stress rapidly increases with increasing strain during the initial stage. This phenomenon results from the competing effect between work hardening and dynamic recovery (DRV). At the stage of low strain, the DRV effect is too weak to overcome the work hardening effect. Therefore, the work hardening effect resulting from dislocation generation and multiplication is significant in this stage, leading to the continuous increase of flow stress. With the further increase of strain, dislocation climbing and sliding is promoted. When strain exceeds the critical strain, dynamic recrystallization (DRX) occurs in the microstructure. The DRV and DRX effects become more influential than the work hardening effect. An instantaneous balance between work hardening and dynamic softening is then obtained when the flow stress reaches its peak value. The annihilation of dislocations leads to the reduction of the work hardening rate. Therefore, the flow stress gradually decreases with the further increase of strain. When the new balance between hardening and softening is obtained, the steady state appears, and no change of flow stress occurs with the further increase of strain.

In addition, the ODS steel possesses positive strain rate sensitivity according to the true strain–stress curves. The flow stress increases with the increase of strain rate and the decline of deformation temperature. The deformation time is reduced with the increase of strain rate, which results in the limited DRV and DRX effect. Therefore, the work hardening effect is promoted, leading to the increase of flow stress. Besides this, the high deformation temperature promotes grain boundary migration and the growth of DRX grains. Moreover, the atomic vibration and diffusion are intense at high temperatures, which is beneficial for dislocation climbing and sliding and thus accelerates the DRV effect [41,42]. That is the reason why the flow stress decreases with the increase of deformation temperature.

#### 3.1.2. Hot Deformation Constitutive Equation

The constitutive equation gives the relationship between the hot deformation temperature and strain rate. Zener and Hollomon [36] have investigated such a relationship and put forward the equation as follows:

(1) $$Z=\dot{\varepsilon }\exp\left(\frac{Q}{RT}\right).$$

where $\dot{\varepsilon }$ is the strain rate, *Q* is the formation activation energy (J/mol), *R* is the gas constant (8.314 J/mol/K), and *T* is the hot deformation temperature (K).

The constitutive behavior is always investigated by empirical equations. Once the strain rate, flow stress, and deformation temperature are known, the constitutive equation can be summarized as follows [43,44,45]:

(2) $$\dot{\varepsilon }=A_{1}\sigma^{n_{1}}\exp\left(-\frac{Q}{RT}\right).$$

(3) $$\dot{\varepsilon }=A_{2}\exp\left(\beta \sigma \right)\exp\left(-\frac{Q}{RT}\right).$$

(4) $$\dot{\varepsilon }=A{\left[\sinh\left(\alpha \sigma \right)\right]}^{n}\exp\left(-\frac{Q}{\mathrm{RT}}\right).$$

where *A*, *A*_1_, *A_2_*, *n*, *n*_1_, α, and β are materials constants, and α can be obtained by α = β/*n*_1_. The powder function depicted in Equation (2) is applicable in low stress conditions. The exponential function shown in Equation (3) is suitable for high stress conditions, and the hyperbolic sine function displayed in Equation (4) is applicative in all stress conditions.

When taking the natural logarithms on both sides of the equation, Equations (2) to (4) can be given as follows:

(5) $$\ln\dot{\varepsilon }=\ln A_{1}+n_{1}\ln\sigma -\frac{Q}{RT}.$$

(6) $$\ln\dot{\varepsilon }=\ln A_{2}+\beta \sigma -\frac{Q}{RT}.$$

(7) $$\ln\dot{\varepsilon }=\ln A+n\ln\left[\sinh\left(\alpha \sigma \right)\right]-\frac{Q}{RT}.$$

**Figure 3:**
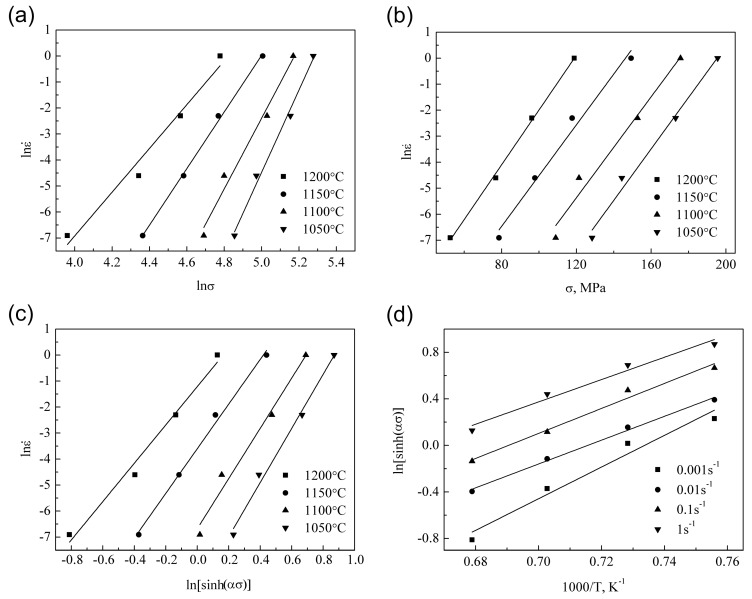
Relationship between the flow stress and strain rate of the ODS sample: (**a**) ln$\dot{\varepsilon }$ vs. ln σ; (**b**) ln$\dot{\varepsilon }$ vs. σ; (**c**) ln$\dot{\varepsilon }$ vs. ln[sinh(ασ)]. Relationship between flow stress and temperature of the ODS sample: (**d**) ln[sinh(ασ)] vs. 1000/T.

The establishment of the constitutive equation is based on the relationship between the peak stress and deformation conditions. It is obvious that *n*_1_, β, and n can be obtained from the slope of the linear regression line in the ln$\dot{\varepsilon }$ vs. ln σ, ln$\dot{\varepsilon }$ vs. ln[sinh(ασ)], and ln$\dot{\varepsilon }$ vs. σ plots. Figure 3a–c give the fitting results of Equations (5)–(7). The fitting results of Equation (7) shown in Figure 3c possess excellent linearly dependent coefficient and parallelism, which means that the hyperbolic sine function is more suitable for the investigation of the constitutive behavior in this work.

The mean value of n (9.0039) is obtained from the slopes of the fitting lines in Figure 3c. Taking a further transformation of Equation (7) gives the following equation:

(8) $$\ln\left[\sinh\left(\alpha \sigma \right)\right]=\frac{\ln\dot{\varepsilon }-\ln A}{n}+\frac{Q}{1000Rn}\times \frac{1000}{T}.$$

As can be seen in Equation (8), when the strain rate is known, Q/(1000Rn) is the slope of the linearly fitting line of the ln[sinh(ασ)] vs. 1000/T plot. The fitting result is illustrated in Figure 3d, and the mean value of Q/(1000Rn) is obtained from the regression result. Since the value of n is determined prior, the value of Q is easily obtained according to Q/(1000Rn). The value of the deformation activation energy (Q) is determined to be 827.526 KJ/mol.

The deformation activation energy Q represents the level of difficulty for the deformation and deformation mechanism. When the deformation activation energy is equivalent to the self-diffusion activation energy, the main deformation mechanism is determined to be DRV. The main deformation mechanism is known to be DRX when the activation energy is far higher than that of the self-diffusion [46,47]. The obtained value of Q is 827.526 KJ/mol under the deformation conditions in this work, far higher than the self-diffusion activation energy in ferrite (239 KJ/mol). Therefore, DRX is the main deformation mechanism in this work, which is consistent with the true strain–stress curves. The deformation activation energy is much higher than that of common high-Cr steel [35]. This phenomenon can be attributed to the addition of nanoparticles in ODS steel. On the one hand, the nanoparticles can effectively prevent the dislocation climbing and sliding, resulting in the reduction of DRV rate. On the other hand, the nanoparticles hinder the formation of new boundaries and boundary migration, which consequently impedes the DRX process [6,14,16]. The nanoparticles in ODS steel play a key role in the strengthening effect, and the deformation activation energy differences between ODS steel and common high-Cr steel are obtained in turn.

The following equation can be obtained by substituting Equation (4) into Equation (1).

(9) $$Z=A{\left[\sinh\left(\alpha \sigma \right)\right]}^{n}.$$

Taking the natural logarithms on both sides of Equation (9) yields the following equation.

(10) lnZ=lnA+nln[sinh(ασ)].

**Figure 4:**
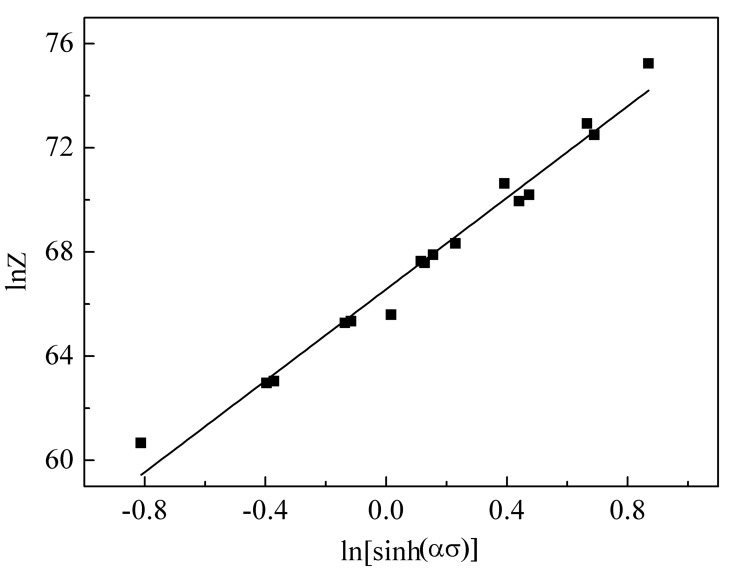
Relationship between the Zener–Hollomon parameter and the flow stress of the ODS sample.

Obviously, ln A and n are the intercept and slope of the linearly fitting line of the ln Z vs. ln[sinh (ασ)] plot, respectively. The linear regression result is presented in Figure 4, and the value of the intercept and slope are 66.571 and 8.7843. According to the analysis mentioned above, the constitutive equation of the 14Cr ODS steel is determined as follows:

(11) $$\dot{\varepsilon }={\left[\sinh(0.0082\sigma_{p})\right]}^{8.7843}\exp(66.571-\frac{827526}{RT}).$$

#### 3.1.3. Processing Map

Prasad and Gegel have considered the hot deformation procedure as a closed thermodynamic system, and the dynamic materials model (DMM) has been proposed based on this hypothesis [48,49]. The processing map can be drawn according to the DMM theory. The microstructure evolution mechanism of the material under different deformation temperatures and strain rates is reflected on the processing map. The processing map is capable of providing the theoretical basis for the selection of parameters during the practical production process [47].

According to the DMM theory, the power dissipation can be expressed as follows [48,50]:

(12) $$P=\sigma \cdot \dot{\varepsilon }=\int_{0}^{\dot{\varepsilon }}\sigma d\dot{\varepsilon }+\int_{0}^{\sigma }\dot{\varepsilon }d\sigma =G+J.$$

where *G* is the power dissipation caused by plastic deformation, and *J* is the power dissipation caused by microstructure evolution.

In the linear dissipative process, *m* = 1, *J* can be expressed as follows:

(13) $$J=J_{\max}=\frac{\sigma \times \dot{\varepsilon }}{2}.$$

In the nonlinear dissipative process, *m* < 1, *J* can be expressed as follows:

(14) $$\eta =\frac{J}{J_{\max}}=\frac{2m}{m+1}.$$

The efficiency of power dissipation *η* is capable of reflecting the microstructure evolution to some extent. The higher the value of *η* is, the higher the value of *J* is. This means that DRV and DRX more easily occur under deformation conditions with higher values of *η*.

Ziegler has put forward the instability criterion and expressed it as [51]

(15) $$\xi \left(\dot{\varepsilon }\right)=\frac{\partial \ln\left(m/\left(m+1\right)\right)}{\partial \ln\dot{\varepsilon }}+m<0.$$

The thermal deformation region can be determined as the instable domain when ξ$\left(\dot{\varepsilon }\right)$ < 0. If the formation rate of system entropy is lower than the strain rate, rheological instability would occur. The formation of shear band, local plastic deformation, and contortion means the appearance of rheological instability [52].

**Figure 5:**
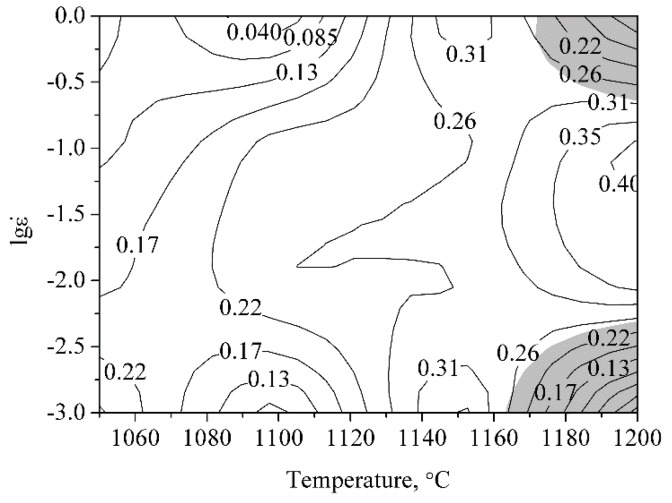
Processing map of the ODS sample at a true strain of 0.5.

The processing map can be drawn on the basis of the analysis above, and the processing map at the true strain of 0.5 is given in Figure 5. There are two instable domains in the processing map. The samples deformed at 1200 °C with the strain rates of 1 and 0.001 s^−1^ are in the instable domains. Samples deformed under other deformation conditions are in the stable domains.

### 3.2. Microstructure Evolution

#### 3.2.1. Effects of Temperature on Microstructure Evolution

**Figure 6:**
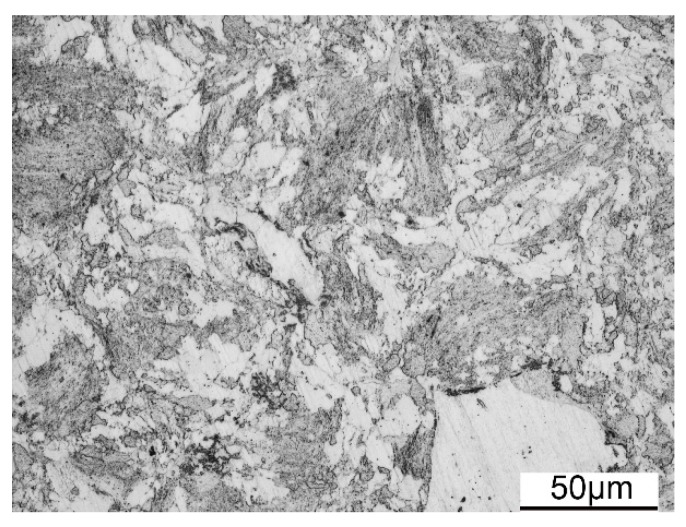
Optical microscope (OM) image of the as-HIPed ODS sample.

The OM image of the as-HIPed ODS steel is displayed in Figure 6. Both large and small grains exist in the microstructure of the as-HIPed ODS steel. The grains in the bright areas are of large size, and the grains in the dark areas are of small size. This bimodal structure has been investigated in previous studies [31,32]. The bimodal structure is the preferable structure in many materials in order to better balance the strength and ductility [31,32,53,54,55,56]. The generation of the bimodal structure in the ODS steel is mainly attributed to the inhomogeneous grain size of the pre-alloyed powders. The stored energy resulted from mechanical alloying is different among the deformed grains, which results in the differences in recovery and recrystallization degrees during the consolidation process [57].

**Figure 7:**
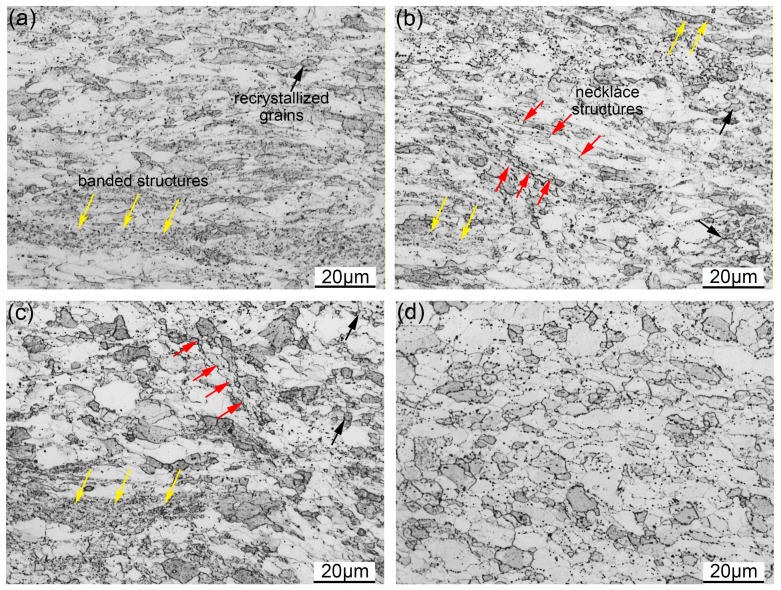
OM images of the samples deformed with the strain rate of 0.01 s^−1^ at (**a**) 1050 °C; (**b**) 1100 °C; (**c**) 1150 °C; (**d**) 1200 °C.

Figure 7a–d illustrate the OM images of the samples deformed with the strain rate of 0.01 s^−1^ at 1050 to 1200 °C. As shown in Figure 7a, the ferrite grains of the ODS steel are stretched into a banded structure normal to the compression direction. Some of the recrystallized grains can be seen in Figure 7a, and the banded structure can also be found. The sample in Figure 7a was deformed at 1050 °C with the strain rate of 0.01 s^−1^. The deformation temperature is low. Therefore, the atomic diffusion rate is too low to efficiently promote grain boundary migration. The microstructure under such deformation conditions is relatively stable, which results in the microstructure illustrated in Figure 7a. DRV is the main deformation mechanism during the deformation process. When the deformation temperature increases to 1100 °C, the amount of recrystallized grains increases, and the necklace structure appears in the microstructure, as can be seen in Figure 7b. The DRX nuclei are generated on the grain boundaries and distributed along the elongated grains. The increase of temperature promotes atomic diffusion, and the recrystallization process is accelerated, leading to the increasing quantity of recrystallized grains, but the initial grains can still be seen in Figure 7b. Figure 7c depicts the microstructure deformed at 1150 °C. Recrystallized grains are the main grains in the microstructure. The recrystallization process is obviously promoted when compared with the microstructure deformed at 1050 and 1100 °C. The fully recrystallized microstructure is obtained after deformation at 1200 °C, which is illustrated in Figure 7d. According to the analysis, the microstructure is sensitive to the deformation temperature under the same strain rate. The diffusion of atomics is easier at higher temperatures, which effectively promote the recrystallization process [41,42]. The quantity of recrystallized grains then increases with the increasing deformation temperature.

#### 3.2.2. Effects of Strain Rate on Microstructure Evolution

**Figure 8:**
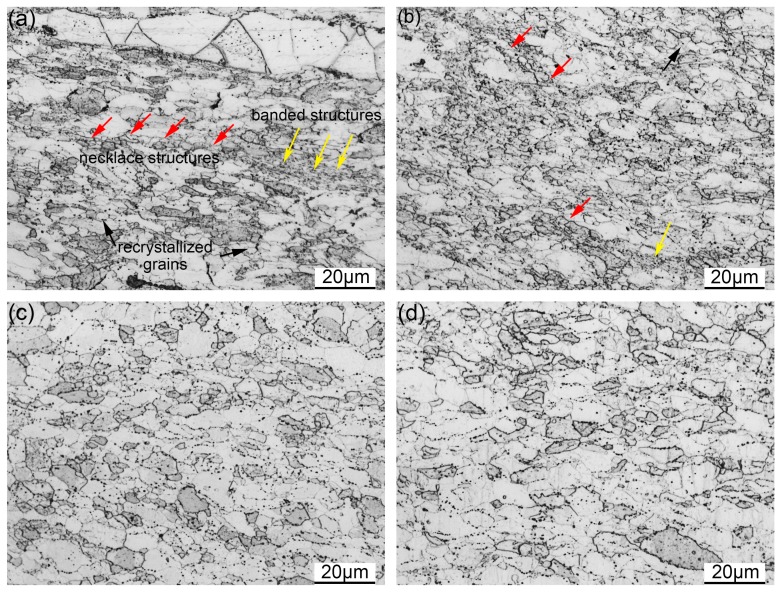
OM images of the samples deformed at 1200 °C with the strain rate of (**a**) 1 s^−1^; (**b**) 0.1 s^−1^; (**c**) 0.01 s^−1^; (**d**) 0.001 s^−1^.

Figure 8 gives the OM images of the samples deformed at 1200 °C with the strain rate ranging from 1 to 0.001 s^−1^. Figure 8a illustrates the microstructure of the sample deformed with the strain rate of 1 s^−1^. The recrystallized grains are distributed inside the elongated grains and along the grain boundaries. Moreover, some unrecrystallized grains can also be seen. The recrystallization process is obviously promoted when the strain rate decreases to 0.1 s^−1^, as depicted in Figure 8b. Most of the recrystallized grains are of small size and distributed around the large grains. The reduction of the strain rate provides more time for the recrystallization process, leading to generation of more DRX nuclei [39,41,42]. Therefore, more small recrystallized grains can be seen in Figure 8b. The fully recrystallized microstructure is obtained with a further decrease of strain rate, as can be seen in Figure 8c,d. The morphology and size of the recrystallized grains are homogeneous after deformation with the strain rates of 0.01 and 0.001 s^−1^. The recrystallization phenomenon is sensitive to the strain rate of deformation. The storage energy is higher during deformation with a faster strain rate, while the deformation time is too short to promote the occurrence of recrystallization. The slower strain rate of deformation is able to provide enough time for recrystallization [39].

**Figure 9:**
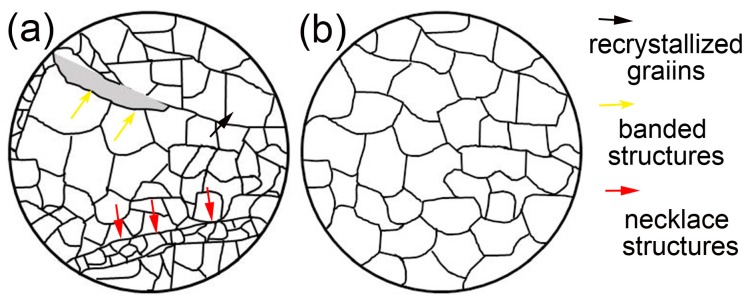
Schematic diagram of the classified microstructures: (**a**) partially recrystallized; (**b**) fully recrystallized.

According to the analysis of deformed microstructures at different deformation temperatures and strain rates, the microstructures can be classified as a partially dynamic recrystallized microstructure and a fully dynamic recrystallized microstructure. The schematic diagram of the classified microstructures is depicted in Figure 9. Figure 9a presents the diagrammatic sketch of the partially dynamic recrystallized microstructure. It consists of the recrystallized grains, banded structure, and the regions including the initial grains in the undeformed sample. The schematic diagram of the fully dynamic recrystallized microstructure is given in Figure 9b. This kind of homogeneous microstructure is composed of recrystallized grains.

#### 3.2.3. Substructures in the Deformed Microstructure

**Figure 10:**
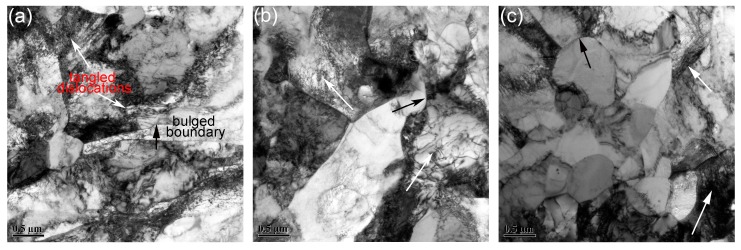
Substructures in the samples deformed with strain rate of 0.01 s^−1^ at (**a**) 1050 °C; (**b**) 1100 °C; (**c**) 1150 °C.

Figure 10 shows the substructures in the samples deformed with strain rate of 0.01 s^−1^. The deformation temperatures in Figure 10a–c are 1050 °C, 1100 °C, and 1150 °C, respectively. The areas marked by black arrows in Figure 10 are bulged boundaries, and the areas marked by white arrows are tangled dislocations. The bulged boundaries tend to generate along the elongated grains, whereas the tangled dislocations are always distributed inside the large grains. The bulged boundaries are related to the energy difference between the adjacent grains, and are known as the nucleation of DRX via discontinuous dynamic recrystallization (DDRX) [58,59]. The formation of bulged boundaries is considered as the symbol of strain-induced grain boundary migration [58,59,60]. The strain difference between the adjacent grains is due to the disparity of dislocation density between them. The grain boundary prefers to bow out to the side of high dislocation density, which can be seen in Figure 10. As marked by black arrows, the side to which the boundary bows out possesses a large amount of dislocations. Most of the dislocations in the microstructures shown in Figure 10 are distributed in the interior of large grains. Some cell structures are generated via dislocation climbing and sliding, and the large grains are divided into several subgrains. Many researchers have investigated the recrystallization mechanism in many materials, and two types of recrystallization mechanisms have been put forward: DDRX and continuous dynamic recrystallization (CDRX) [61,62]. Formation of subgrains is indicative of CDRX. The dislocation cell boundaries would transform into low-angle subgrain boundaries and then into high-angle boundaries. Both characteristics of DDRX and CDRX can be seen in Figure 10, meaning double recrystallization mechanisms performed during deformation.

**Figure 11:**
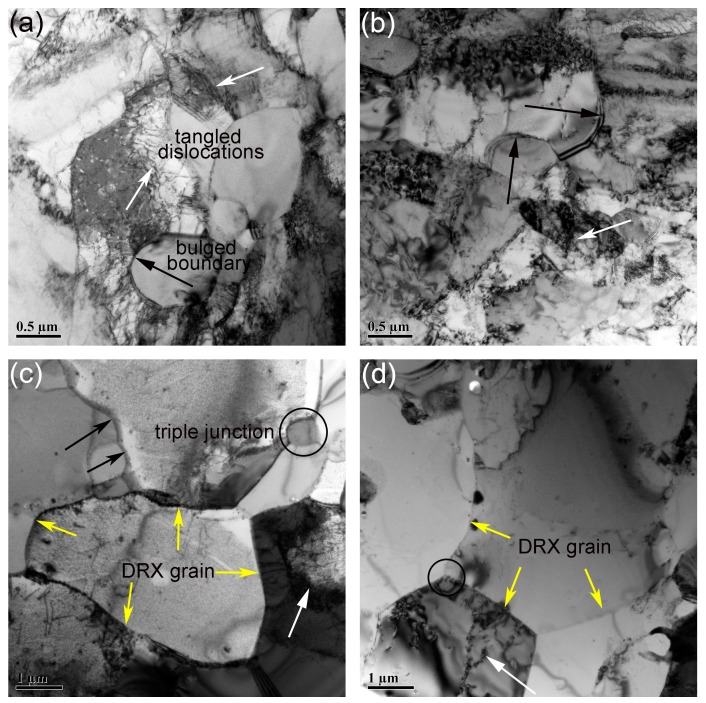
Substructures in the samples deformed at 1200 °C with strain rate of (**a**) 1 s^−1^; (**b**) 0.1 s^−1^; (**c**) 0.01 s^−1^; (**d**) 0.001 s^−1^.

Substructures in the samples deformed at 1200 °C with strain rate ranging from 1 to 0.001 s^−1^ are depicted in Figure 11. The areas marked by black and white arrows are bulged boundaries and tangled dislocations, respectively. The areas marked by circles are triple junctions. Figure 11a,b show the substructures in the samples deformed with the strain rate of 1 and 0.1 s^−1^. Similar to the results displayed in Figure 10, both bulged boundaries and tangled dislocations exist in Figure 11a,b. The fraction of recrystallized grains in samples deformed at 1200 °C is higher than that in the samples deformed with strain rate of 0.01 s^−1^, which can be seen in Section 3.2.1 and Section 3.2.2. That is the reason why the overall size of grains is larger than that in Figure 10. The substructures in the samples deformed with the strain rate of 0.01 and 0.001 s^−1^ are illustrated in Figure 11c,d. Only a small quantity of dislocations and bulged boundaries can be seen in the microstructures since the fully dynamic recrystallized grains have been obtained. Bulged boundaries are distributed along the recrystallized grain boundaries, and tangled dislocations are distributed in the interior of the recrystallized grains. Triple junctions also exist in the microstructure. The density of defects at or near the triple junctions is higher than that at neighboring boundaries. Triple junctions thus have excess energy beyond the energy of neighboring boundaries [58]. Therefore, the DRX nuclei tend to generate at triple junctions. A new cycle of recrystallization would occur with the further increase of true strain [5].

Substructures are the symbols of the corresponding recrystallization mechanism. However, TEM results are simply an assistant method for the characterization of the recrystallization mechanism. More analysis such as electron backscatter diffraction (EBSD) should be done in order to identify the recrystallization behavior and recrystallization mechanism during the deformation process [37,58,62]. All we know is that both DDRX and CDRX exist during the deformation process, but which one is the major deformation mechanism still needs further investigation.

#### 3.2.4. Nanoparticles

**Figure 12:**
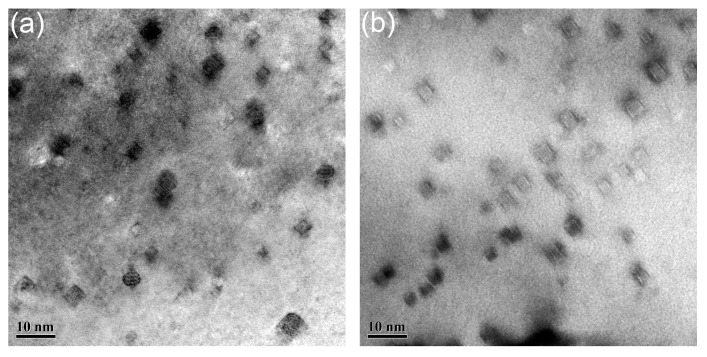

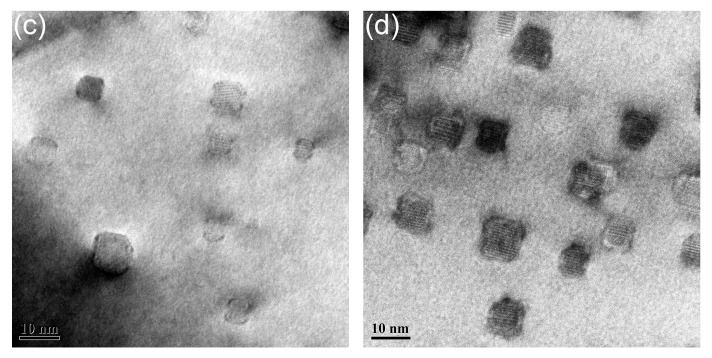
Distribution of nanoparticles in the samples deformed with the strain rate of 0.01 s^−1^ at (**a**) 1050 °C; (**b**) 1100 °C; (**c**) 1150 °C; (**d**) 1200 °C.

The distribution of nanoparticles in the samples deformed with the strain rate of 0.01 s^−1^ at 1050 to 1200 °C is illustrated in Figure 12. Nanoparticles shown in Figure 12 are all with size less than 10 nm, and no obvious morphology evolution can be found with the increasing temperature. The nanoparticles have been identified as Y_2_Ti_2_O_7_ in previous work [31]. These nanoparticles possess three kinds of morphologies: cubic, cuboidal, and chamfered cuboid. The cubic nanoparticles become cuboidal and then chamfered cuboid nanoparticles with the increase of particle size. This phenomenon is related to the elastic energy between the nanoparticles and matrix [63]. The sizes of the nanoparticles are within the range of 5 nm to 10 nm in all the samples shown in Figure 12.

**Figure 13:**
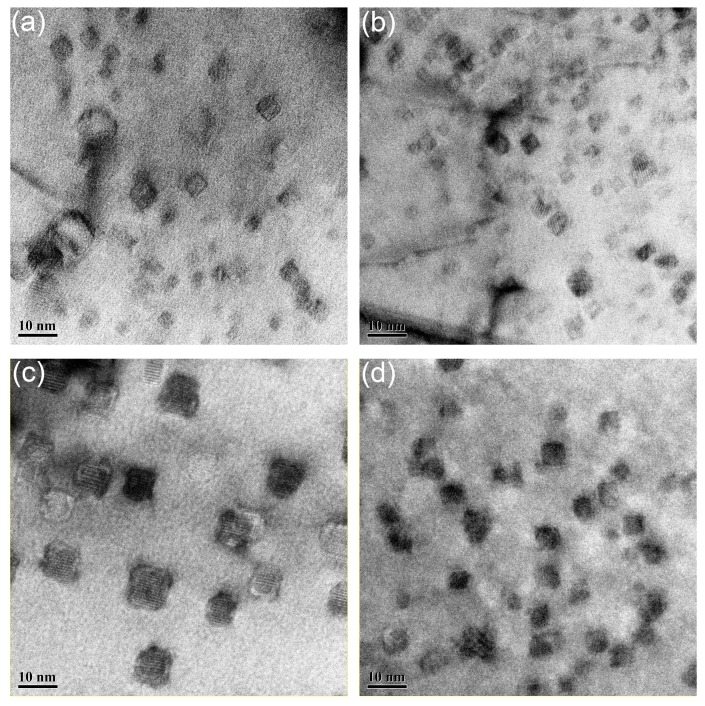
Distribution of nanoparticles in the samples deformed at 1200 °C with strain rate of (**a**) 1 s^−1^; (**b**) 0.1 s^−1^; (**c**) 0.01 s^−1^; (**d**) 0.001 s^−1^.

The average sizes of nanoparticles in the samples deformed at 1150 and 1200 °C are slightly larger than those in the samples deformed at 1050 and 1100 °C, but the morphologies remain unchanged, resulting from the stable interface between the nanoparticles and matrix. Figure 13 gives the distribution of nanoparticles in the samples deformed at 1200 °C with strain rate ranging from 1 to 0.001 s^−1^. Same as in Figure 13, all the nanoparticles possess three types of morphologies, and no obvious increase in the size can be seen.

**Figure 14:**
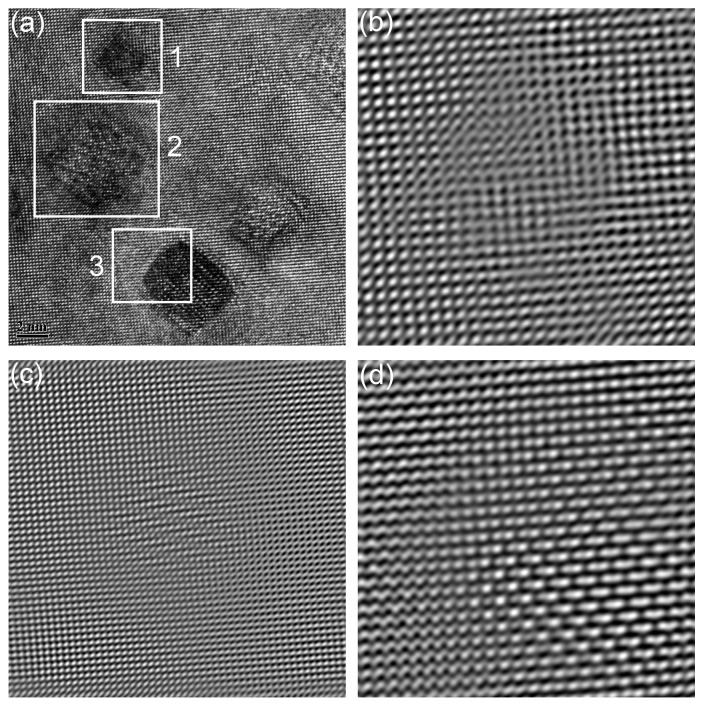
The interfaces between nanoparticles and matrix in the sample deformed at 1200 °C with strain rate of 0.001 s^−1^: (**a**) HRTEM graph of nanoparticles; (**b**–**d**) inverse FFT (IFFT) patterns obtained from the areas marked 1 to 3 in (**a**).

**Figure 15:**
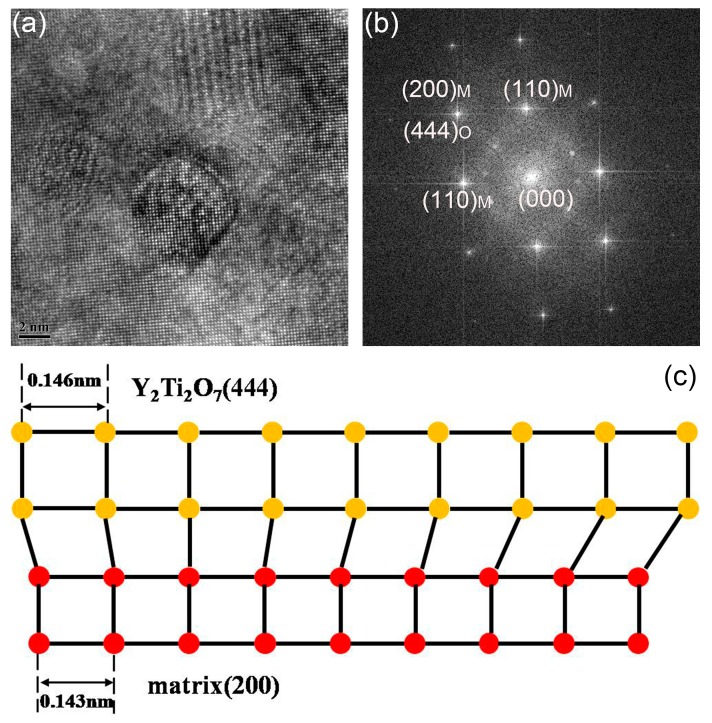
(**a**) HRTEM graph and (**b**) the corresponding FFT pattern of the marked particle in the ODS sample deformed at 1200 °C with the strain rate of 0.001 s^−1^; (**c**) schematic diagram of the coherent interface between the marked nanoparticle and matrix.

The results indicate that nanoparticles with size less than 10 nm are insensitive to the increase of temperature and strain rate. These nanoparticles play an effective role in pinning the dislocation motion and grain boundary migration, due to the excellent stability [10,11]. The deformation temperature is not high enough to promote the growth of such nanoparticles. Moreover, the deformation time is too low to initiate the growth of nanoparticles either. High temperature and long deformation time give potential for the growth of nanoparticles. The interfaces between nanoparticles and matrix in the sample deformed at 1200 °C with strain rate of 0.001 s^−1^ are thus investigated. Results are shown in Figure 14. Figure 14a is the HRTEM graph of nanoparticles. The nanoparticles marked by Arabic numerals are in different morphologies. Particle 1 represents the nanoparticles in cubic morphology. Particle 2 represents the nanoparticles in cuboidal shape, and Particle 3 represents the nanoparticles with the morphology of chamfered cuboid. Figure 14b–d are the inverse fast Fourier transform (IFFT) patterns obtained from the areas marked by rectangles in Figure 14a. Figure 14b–d correspond to Particles 1 to 3, respectively. No misfit dislocations can be seen in the figure, which means that the particles marked in Figure 14a are all coherent with the matrix [63,64]. Figure 15a gives the HRTEM graph of nanoparticles, and the corresponding FFT pattern of the marked particle is shown in Figure 15b. According to the identified result shown in Figure 15b, the (444) plane of Y_2_Ti_2_O_7_ is parallel to the (200) plane of the matrix. The schematic diagram of the coherent interface between nanoparticle and matrix is depicted in Figure 15c. The interplanar spacing of (444) is 0.146 nm, and the interplanar spacing of (200) is 0.143 nm. It is a coherent interface with elastic distortion. Zhong et al. [65] have concluded that the coherency would be lost when the recrystallized microstructure is obtained during annealing, which is different with the results displayed here. The differences may be ascribed to the diverse processing methods. The recrystallized microstructure is obtained via dynamic recrystallization, which needs less time than static recrystallization. The deformation time is too short to change the interface between nanoparticles and the matrix. The coherent relationship between nanoparticles and matrix plays a key role in the dislocation interaction with nanoparticles [66]. The pinning effect of nanoparticles and their ability to hinder dislocation motion and boundary migration strongly depend on the interface property [63,65]. The size, morphology, and interface of the nanoparticles are almost changeless during deformation, which illustrates the stability of nanoparticles. The recrystallized microstructure is obtained without change of the strengthening nanoparticles. Deformation is thus an effective way to control the microstructure of ODS steel.

## 4. Conclusions

1. Deformation of the ODS steel was performed at the temperature range of 1050–1200 °C with the strain rate ranging from 1 to 0.001 s^−1^. The deformation activation energy of the 14Cr ODS steel under these conditions was calculated as 827.526 KJ/mol. The constitutive equation was established and expressed as
  (16) $$\dot{\varepsilon }={\left[\sinh(0.0082\sigma_{p})\right]}^{8.7843}\exp(66.571-\frac{827526}{RT}).$$
2. The processing map at the strain rate of 0.5 is provided as an example, and the deformed microstructures perfectly correspond to the processing map. The processing map provides the theoretical basis for a practical production process.
3. The ODS steel is sensitive to the deformation temperature and strain rate. The fraction of the recrystallized grains increases with the increasing deformation temperature and the reduction of strain rate. Partially and fully dynamic recrystallized microstructures are obtained after deformation under different conditions. The schematic diagrams of these microstructures were established.
4. Large amounts of tangled dislocations and bulged boundaries are found in the partially dynamic recrystallized microstructure. Small amounts of dislocations and bulged boundaries can also be seen in the fully dynamic recrystallized microstructure.
5. The nanoparticles with size less than 10 nm are stable during the deformation process. Both the size and morphology of the nanoparticles remain unchanged after deformation. Nanoparticles with different size and morphology in the sample deformed at 1200 °C with strain rate of 0.001 s^−1^ were investigated, and interfaces between them and the matrix are coherent. Coherency loss does not occur during deformation.
